# Curcumin Supplementation Protects Broiler Chickens Against the Renal Oxidative Stress Induced by the Dietary Exposure to Low Levels of Aflatoxin B1

**DOI:** 10.3389/fvets.2021.822227

**Published:** 2022-01-24

**Authors:** Sara Damiano, Watanya Jarriyawattanachaikul, Flavia Girolami, Consiglia Longobardi, Carlo Nebbia, Emanuela Andretta, Chiara Lauritano, Sihem Dabbou, Giuseppina Avantaggiato, Achille Schiavone, Paola Badino, Roberto Ciarcia

**Affiliations:** ^1^Department of Veterinary Medicine and Animal Productions, University of Naples Federico II, Naples, Italy; ^2^Department of Veterinary Sciences, University of Torino, Grugliasco, Italy; ^3^Department of Mental, Physical Health and Preventive Medicine, University of Campania Luigi Vanvitelli, Naples, Italy; ^4^Ecosustainable Marine Biotechnology Department, Stazione Zoologica Anton Dohrn, Naples, Italy; ^5^Center Agriculture Food Environment (C3A), University of Trento, San Michele all'Adige, Italy; ^6^Institute of Sciences of Food Production, Italian National Research Council, Bari, Italy

**Keywords:** Aflatoxin B1, turmeric powder, curcumin, oxidative stress, kidney, chicken—broiler, renal damage

## Abstract

Aflatoxin B1 (AFB1) causes hepatotoxicity, immunotoxicity, and kidney damage, and it is included in group I of human carcinogens. The European Commission has established maximum limits of AFB1 in feed, ranging from 5 to 20 μg/kg. Chicken is moderately sensitive to AFB1, which results in reduced growth performance and economic losses. Oxidative stress triggered by AFB1 plays a crucial role in kidney damage and the antioxidant activity of Curcumin (CURC) could help in preventing such adverse effect. Twenty-days-old broilers were treated for 10 days with AFB1 (0.02 mg/kg feed), alone or in combination with CURC (400 mg/kg feed), to explore the effects on the renal tissue. Animals exposed to AFB1 alone displayed alterations of the oxidative stress parameters compared with controls: serum antioxidant capacity, and enzymatic activity of kidney superoxide dismutase, catalase and glutathione peroxidase were decreased, while renal malondialdehyde levels and NADPH oxidase complex expression were increased. The administration of CURC attenuates all the oxidative stress parameters modified by AFB1 in the chicken kidney, opening new perspectives in the management of aflatoxicosis.

## Introduction

Foodstuffs, grains and feeds are the ideal substrates for the growth of mycotoxigenic fungi. Mycotoxins are fungal secondary metabolites that can accumulate in these resources leading to important economic losses, as well as to problems for livestock and human health (1–3).

Owing to climate change, mycotoxigenic *Aspergillus* species have spread, increasing the risk of feed and food contamination worldwide (4, 5). Such condition is particularly evident in the Mediterranean area due to the increase of average temperature, CO_2_ levels and drought episodes, alternating with heavy precipitations, especially during the summer season (6). The major mycotoxins produced by *Aspergillus* spp. are aflatoxins (AFs), and namely B1, B2, G1, and G2. From a chemical point of view, these are all furanocoumarins and Aflatoxin B1 (AFB1), produced by *A. flavus*, is the most studied among them. AFB1 is well-known for its carcinogenic effects, and it is classified in group I of the human carcinogenic compounds (7). It also causes hepatotoxicity (8), kidney and heart damage (9), immunotoxicity (10), and could lead to fatal consequences in both farm animals and humans (11). Hence, most countries all over the world have set regulatory limits for AFs in both food and feed. In the European Union the maximum permitted level range for complementary and complete feed for livestock is 0.005 (dairy ruminants) – 0.02 mg/kg (12).

Poultry farming is one of the most important segments of agro-industries and it can be at a high risk of negative effects in case of AFB1 feed contamination, which could result in low performance and decreased quality of both eggs and meat, with negative economic feedback (13, 14).

*In vivo* studies performed in both laboratory and farm species have demonstrated the capability of AFB1 to induce oxidative stress in several organs and tissues, including the kidney (9, 15, 16). Such effects are associated with a strong lipid peroxidation and a concomitant decrease in the antioxidant enzymes involved in the detoxification of superoxide anions and peroxides. The oxidative stress caused by AFB1 plays a crucial role also in chicken kidney damage (17). Many mitigation/detoxification methods (e.g., post-harvest physical processing operations, chemical agents, mycotoxin binders) to remove or to reduce the mycotoxin contamination of food and feed have been described so far (18, 19). However, none of them is completely effective (20). In the last few years, many studies have engaged in the search for eco-friendly dietary supplements, which could prevent or reduce the oxidative stress induced by mycotoxins. For instance, the supplementation of vitamins has showed antioxidant and anti-inflammatory effects in poultry birds (21, 22).

Curcumin (CURC), a polyphenolic compound which belongs to the curcuminoid group, has several pharmacological effects, including anti-inflammatory, antioxidant, radioprotective, and tissue protective activity in both experimental animals and humans (23), making it suitable as a potential chemo-preventive agent against the tissue damage induced by AFB1. The protective role of CURC against AFB1-induced oxidative stress, acting as a scavenger for ROS and DNA damage, has been reported in different species (23, 24). However, the exact mechanism of CURC regulation of oxidative stress is not clear yet.

In the present work, we investigated the benefits of the dietary supplementation with CURC from turmeric powder in broilers exposed to AFB1 at concentrations approaching the EU maximum permitted limits. Thus, the main purposes of our research were: (1) to explore the AFB1-induced oxidative stress in the renal tissue; (2) to test the ability of CURC in counteracting such effects; and (3) to determine the involvement of the nicotinamide adenine dinucleotide phosphate (NADPH) oxidase (NOX) in the AFB1-mediated damage and its possible modulation by the antioxidant. Indeed, NOX appears to be the most important contributor to ROS generation in the renal tissue (25, 26) and has a central role in the development of kidney disease (27). Evaluated parameters included: the enzymatic activities of renal superoxide dismutase (SOD), catalase (CAT) and glutathione peroxidase (GPx), the malondialdehyde (MDA) levels and NOX expression in kidney tissue, and the serum antioxidant capacity (SAC).

## Materials and Methods

### Animals, Diets, and Experimental Design

The *in vivo* study was approved by the Institutional Animal Care and Ethic Committee of the University of Turin (Approval number = 319508/2017-PR) and conducted at the poultry facility of the University of Turin located in Carmagnola (TO), Italy. A total of 32 male broiler chickens (ROSS 308) 18-days-old and 751.88 ± 46.28 g of weight was housed in cages (according to Directive 2007/43) and received a standard basal diet (210 g/kg of crude protein; 13.6 MJ/kg of Metabolizable Energy) *ad libitum*. After 4 days of adaptation period (22-days-old), they were randomly distributed over 16 cages (2 birds per cage). The cages (60 × 60 cm) were placed in an insulated room with devices to control the temperature, light and humidity. Each cage had a linear feeder at the front and a nipple drinker at the back. The animals were divided into four experimental groups (8 animals per-treatment): Ctr group (basal diet, BD); AFB1 group (BD + 0.02 mg/kg feed AFB1); CURC group (BD + 400 mg/kg feed CURC); and AFB1 + CURC group (BD + 0.02 mg/kg feed AFB1 + 400 mg/kg feed CURC). An artificially contaminated feed containing ~0.02 mg/kg of AFB1 (concentration equal to the limit set by the Reg. CE No. 574/2011 for complementary and complete feed for poultry) was prepared by spiking a blank feed with a fungal culture extract. Briefly, a corn culture (0.5 kg) of *A. flavus* (ITEM Microbial Culture Collection of ISPA No. 7828, http://www.ispa.cnr.it/Collection) was prepared and incubated in the dark at 25°C for 1-month. Then the fungal culture was ground, dried (60°C) and extracted with a water/acetonitrile (70/30, v/v) mixture. The mean concentrations (mean ± SD, *n* = 3) of AFB1 and AFB2 in the culture extract, determined by an Ultra Performance Liquid Chromatography (UPLC) system coupled with a fluorometric detector (FD), was 10.0 ± 0.1 and 0.70 ± 0.02 μg/mL, respectively. To prepare 20 kg of the artificially contaminated feed, an appropriate volume of the culture extract (40 mL) containing 0.4 mg of AFB1 and 0.028 mg of AFB2 was mixed with 1 kg of a commercial blank corn. The corn sample was mixed in the BD (20 kg) to obtain the desired level of AFs. Final AF content was checked on 5 feed samples by UPLC-FD according to the ISO standard method 17375:2006. The average amount of AFs in the artificially contaminated diet was 18.8 ± 3.7 μg/kg of AFB1 and 1.4 ± 0.2 μg/kg of AFB2 (mean ± SD, *n* = 5), and it can be considered as homogeneous. The amount of AFB1 and AFB2 in the BD used for the Ctr group was 5.0 ± 1.3 μg/kg and 0.9 ± 0.1 μg/kg, respectively (mean ± SD, *n* = 3). No other major mycotoxin (i.e., ochratoxin A, fumonisins, deoxynivalenol, T-2 toxin, HT-2 toxin, and zearalenone) was detected in the BD at levels higher than the LOD of the analytical method (28). CUR was added to the basal diet in the form of food grade turmeric powder (*Curcuma longa*) (Biorama, Rogeno—LC, Italy), containing 2.5% curcumin, desmethoxycurcumin, and bis-desmethoxycurcumin (85/10/5), to reach the concentration of 400 mg/kg feed, according to previous studies (29, 30). To allow homogeneity in the feed, CURC was dissolved in soybean oil (0.6 kg) prior to mixing all feed ingredients. An equal amount of oil was added to the diet of each experimental group. The treatment lasted 10 days, from 23 to 32 days of age. Health status and mortality were monitored daily throughout the whole experimental period: no signs of illness were observed, and the mortality rate was zero in all the groups. Blood samples from each animal were collected from the brachial vein at the beginning (T0) and at the end (T10) of the trial. At the end of the experiment, broilers were sacrificed by an intravenous overdose of sodium pentobarbital, and both kidneys from each animal were removed. Specimens from each kidney were pooled into aliquots corresponding to a single animal immediately frozen in liquid nitrogen and stored at −80°C for the following analyses.

### Renal SOD, CAT, and GPx Activity Assays

The total activity of SOD, CAT, and GPx was evaluated in kidney homogenates by the Glomax Multi detection system spectrophotometer (Promega Corporation, Madison, WI, USA) according to our previously reported protocol (31). Total protein content of each sample was measured by the BCA Protein Assay Kit (Bio-Rad, Milan, Italy), and data were expressed as units (U) per milligrams of protein.

### Evaluation of Serum Antioxidant Capacity

The SAC was measured by the OXY-Adsorbent test from Diacron (Grosseto, Italy). The assay evaluates the ability of the serum barrier to neutralize the oxidative action induced by an hypochlorous acid solution (HClO). The method was modified from Jansen and Ruskovska (32). Briefly, 2 μl of serum samples diluted 1:100 (v/v) with MilliQ-water were incubated with 200 μl of HClO solution for 10 min at 37°C in a 96-well-plate. Then, 2 μl of chromogenic reagent (N, N-diethyl-p-phenylenediamine) was added. The intensity of the colored complex is inversely related to the total antioxidant capacity in serum. The absorbance values were measured at 505 nm using the Glomax Multi detection system spectrophotometer. Data were expressed as mmol of HClO per mL.

### Thiobarbituric Acid Reactive Substances Assay

Kidney samples were disrupted and homogenized using TissueLyser LT (Qiagen, Hilden, Germany) for 5 min at 50 Hz, in 200 μl of NaCl (0.9% w/v), 200 μl of TCA (10% w/v), and 4 μl of BHT (2% w/v). After centrifugation at 13,000 × g for 15 min at 4°C, the supernatants were collected and transferred to fresh tubes and kept on ice to avoid oxidation effects until analysis. The thiobarbituric acid reactive substances (TBARS) assay was modified from Espin et al. (33). The TBA solution was composed of 15% of trichloroacetic acid (TCA) (w/v) in glacial acetic acid, 0.38% of 2-thiobarbituric acid (TBA; w/v), and 0.25 N of hydrochloric acid. Finally, the TBA solution was adjusted to the finale volume with MilliQ-water. All samples, blank, and/or MDA standard solutions were mixed with 400 μl of TBA solution and then vortexed for 1 min. The formation of MDA-TBA adducts was obtained by placing samples in a water bath for 60 min at 95°C. The reaction was stopped by cooling in a cold-water bath for 10 min, and then samples were centrifuged at 13,000 × g for 15 min. The absorbance values were measured at 532 nm using the spectrophotometer. All samples, blank, and the MDA standard curve were done in triplicate. Data were expressed as nmol of MDA per mg of tissue.

### Evaluation of Serum DNA Damage

The serum levels of 8-hydroxy-2′-deoxyguanosine (8-OHdG), as a marker of DNA damage, were evaluated by an ELISA commercial kit from StressMarq (Biosciences INC, Victoria, BC, Canada), using the spectrophotometer Glomax Multi Detection System. Data were expressed as ng of 8-OHdG per mL.

### RNA Extraction, Complementary DNA Synthesis, and Quantitative Real-Time PCR (qRT-PCR)

Total RNA from kidney tissue was isolated using TRIzol™ Reagent (Invitrogen, Waltham, MA, USA), and treated with DNase, according to the manufacturer's protocol. RNA quantity and purity was assessed by NanoDrop (ND-1000 UV–Vis spectrophotometer; NanoDrop Technologies, Wilmington, NC, USA). For gene expression analysis, 1 μg of total RNA for each sample was retrotranscribed into complementary DNA (cDNA) with the iScript^TM^ cDNA Synthesis Kit (BIORAD, Hercules, CA, USA), following the manufacturer's instructions, using the GeneAmp PCR System 9700 (Perkin Elmer, Waltham, MA, USA). Primers for NOX4 (gene of interest) and GAPDH (reference gene) were designed using the software Primer3 v. 0.4.0 [http://frodo.wi.mit.edu/primer3/; (34)] and the identity of each sequence was confirmed using the blastn function in the bioinformatics tool BLAST (Basic local alignment search tool; https://blast.ncbi.nlm.nih.gov/Blast.cgi). The used primer sequences were: NOX4 (fw) GGAGTGCTCAAGTACCAGACC; NOX4 (rev) AGTGCGACTGGAACTTGGG; GAPDH (fw) AGGCGAGATGGTGAAAGTCG; GAPDH (rev) CCGTTCTCAGCCTT-GACAGT. qRT-PCR thermal profile and procedure were according to Lauritano et al. (35). Briefly, all qRT-PCR reactions were done in triplicates to reduce the intra-assay variability and included three no-template negative controls (NTC) for each primer pair. The expression levels analysis was performed by Excel-applet qGene software.

### Western Blot Analysis

Kidney samples were homogenized in RIPA buffer with a protease inhibitor mix (complete™, Mini, EDTA-free Protease Inhibitor Cocktail Tablets, Roche, Basel, Switzerland), employing the TissueLyser LT to promote lysis. Total protein content of each sample was measured by the BCA Protein Assay Kit. Mini-PROTEAN® precast gel 4–12% (Bio-Rad, Milan, Italy) was operated for loading the samples and Precision Plus Dual Color Standards (Bio-Rad, Milan, Italy) was used as molecular weight marker. Trans-Blot® Turbo Nitrocellulose membrane (Bio-Rad, Milan, Italy) was employed to transfer proteins. In order to detect the oxidative damaged proteins in the samples, the primary antibody anti-NOX4 (1:1,000, Rabbit polyclonal antibody, Abcam, Milan, Italy) was used. Anti-GAPDH (1:20,000, Rabbit monoclonal antibody, OriGene, Herford, Germany) antibody was used as housekeeping expression protein. Nitrocellulose membranes were incubated with HRP-labeled secondary antibodies (1:2,000, Santa Cruz Biotechnology, Heidelberg, Germany) according to the species of primary anti-bodies and bands were detected using Clarity Western ECL substrate (Bio-Rad, Milan, Italy). Relative signal intensities were quantified by ChemiDoc™ Imaging System (Bio-Rad, Milan, Italy) with the Bio-Rad Quantity One® software version 4.6.3. and normalized in relation to GAPDH bands.

### Statistical Analysis

Data were expressed as mean ± standard deviation (SD). Each animal group consisted of 8 broiler chickens and normal distribution of data was assessed according to the D'Agostino and Pearson normality omnibus test. Significant differences among groups were evaluated by one-way analysis of variance (ANOVA), followed by the Tukey's *post-hoc* tests. Differences were considered statistically significant when the two-sided *p* < 0.05. Data analysis was performed with the GraphPad Prism 7.03 software (Graph Pad Software, San Diego, CA, USA).

## Results

During the trial all birds remained healthy, and no mortality was observed. The average daily feed intake was not influenced by the dietary treatment and ranged between 131.7 g/day/bird and 145.1 g/day/bird.

### Effects of CURC and AFB1 on Renal SOD, CAT, and GPx Enzymatic Activity

**Figure 1:**
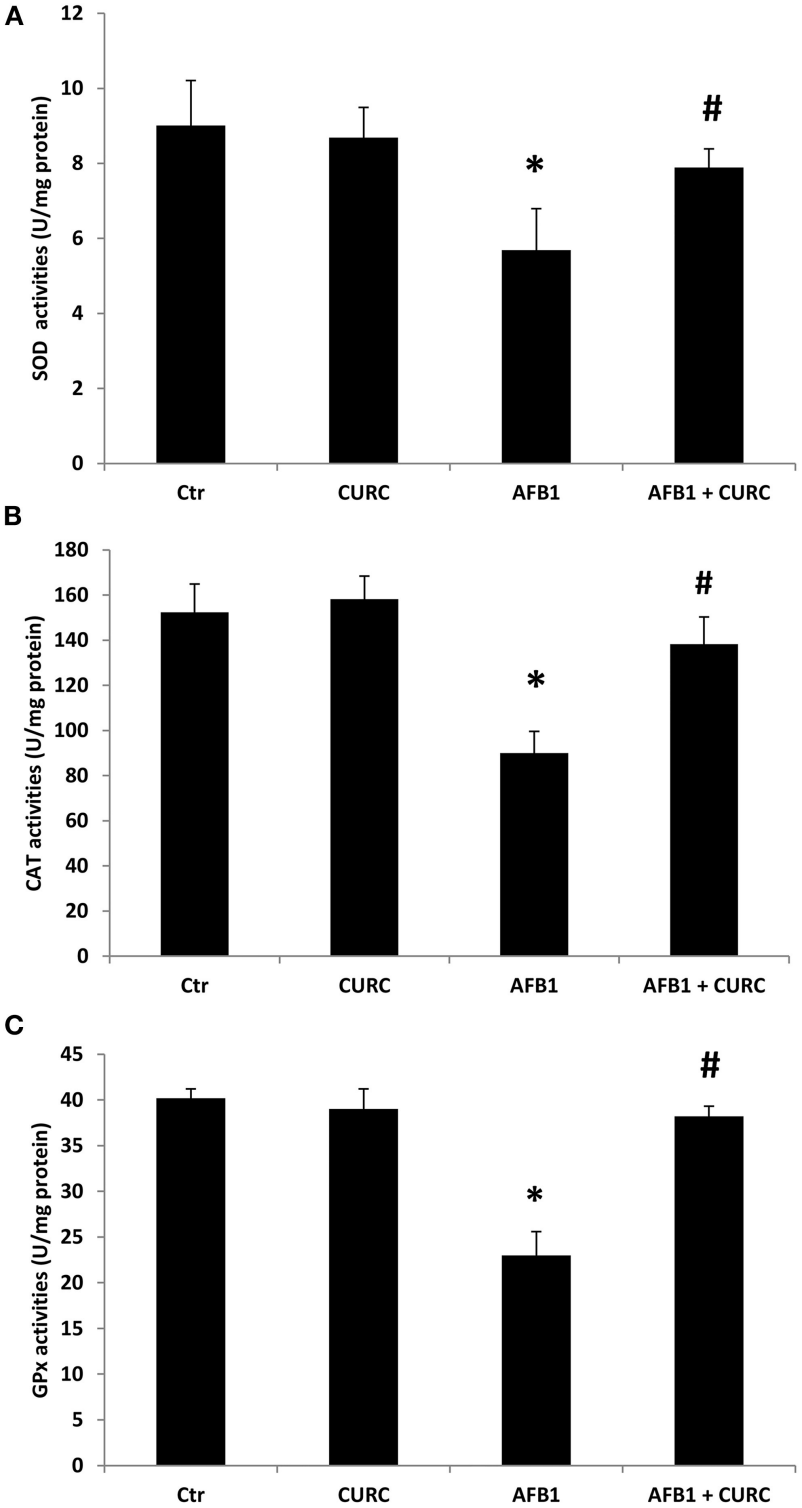
Effects of Curcumin (CURC) and Aflatoxin B1 (AFB1) on superoxide dismutase (SOD), catalase (CAT), and glutathione peroxidase (GPx) activities in broiler renal tissue at the end of treatment (T10). **(A)** Renal SOD activity, **(B)** renal CAT activity, **(C)** renal GPx activity. Ctr, control group; CURC, Curcumin group; AFB1, Aflatoxin B1 group; AFB1 + CURC, Aflatoxin B1 group plus Curcumin. Data are expressed as mean ± standard deviation (SD), *n* = 8 (*p < 0.05 vs. Ctr; ^#^*p* < 0.05 vs. AFB1).

The activity of the three main antioxidant enzymes (i.e., SOD, CAT, and GPx) has been assessed in the renal tissue of the experimental groups after 10 days of treatment. As shown in Figure 1, animals exposed to AFB1 alone displayed a significant reduction of SOD, CAT and GPx activity up to 40% compared to controls (^*^p < 0.05 AFB1 vs. Ctr). CURC supplementation in the absence of AFB1 did not affect the activity of any of the investigated enzymes. However, CURC was able to almost completely counteract the impairment of SOD, CAT, and GPx induced by AFB1 (^#^*p* < 0.05 AFB1 vs. AFB1 + CURC), restoring their activity to normal values.

### Effects of CURC and AFB1 on Serum Antioxidant Capacity and Renal Lipid Peroxidation

**Figure 2:**
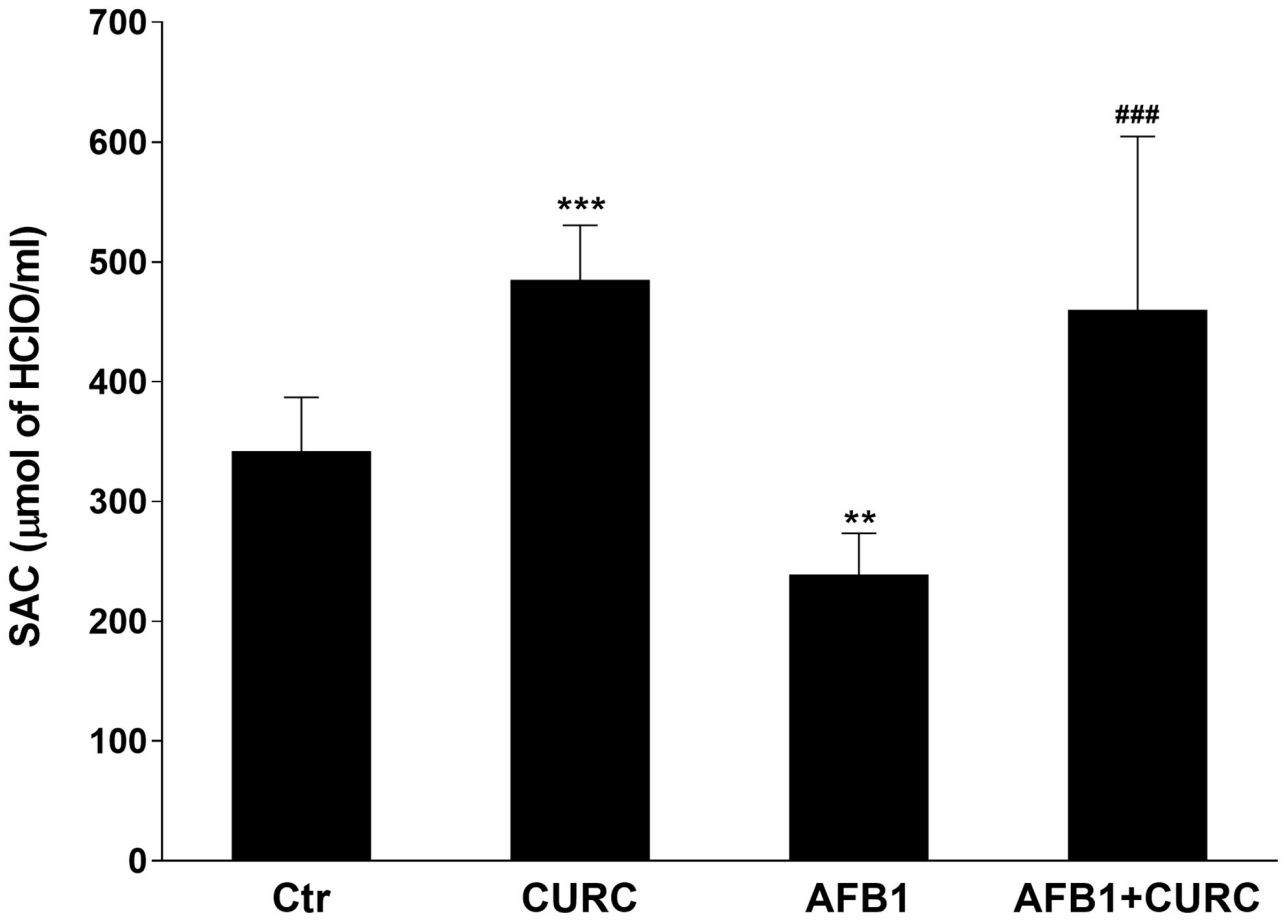
Effects of Curcumin (CURC) and Aflatoxin B1 (AFB1) on serum antioxidant capacity (SAC) in broilers at the end of treatment (T10). Ctr, control group; CURC, Curcumin group; AFB1, Aflatoxin B1 group; AFB1 + CURC, Aflatoxin B1 group plus Curcumin. Data are expressed as mean ± standard deviation (SD), *n* = 8 (**p < 0.01, ***p < 0.0001 vs. Ctr; ^###^*p* < 0.001 vs. AFB1).

The broiler antioxidant defense system has been checked through the measurement of SAC at the beginning and at the end of the experiment. Before the administration of the different diets, the levels of SAC were equivalent among groups (data not shown). The exposure for 10 days to AFB1 alone triggered a statistically significant reduction of SAC by ~30% in comparison with control animals (^**^p < 0.01 AFB1 vs. Ctr). On the contrary, CURC supplementation significantly increased broiler SAC with respect to the controls up to 25% (^***^p < 0.0001 CURC vs. Ctr). Likewise, the negative effect induced by AFB1 was counteracted by the concomitant supplementation with CURC that restored SAC to control values (^###^*p* < 0.0001 AFB1 vs. AFB1 + CURC) (Figure 2).

**Figure 3:**
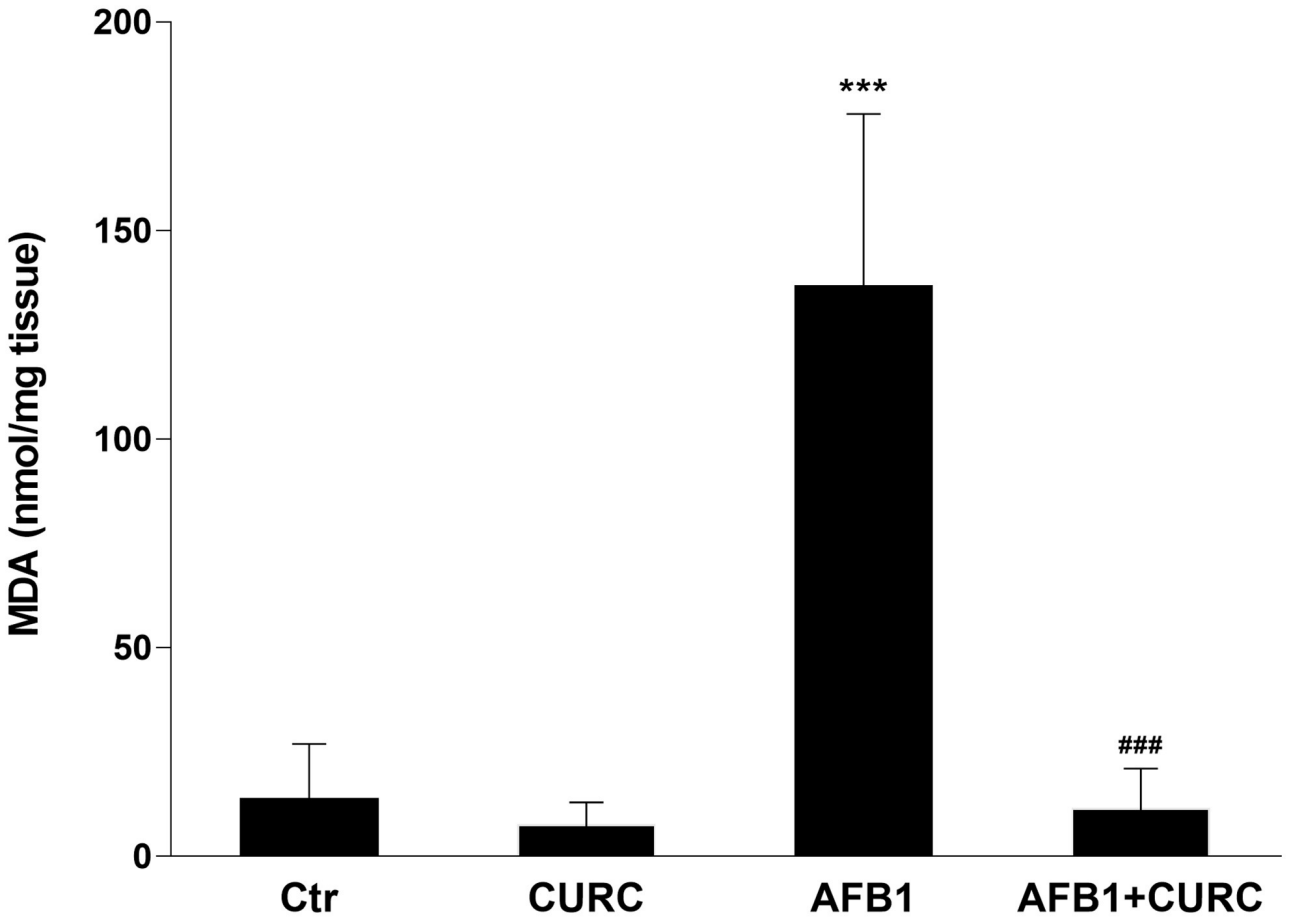
Effects of Curcumin (CURC) and Aflatoxin B1 (AFB1) on renal lipid peroxidation, measured as MDA levels, in broilers at the end of the treatment (T10). Ctr, control group; CURC, Curcumin group; AFB1, Aflatoxin B1 group; AFB1 + CURC, Aflatoxin B1 group plus Curcumin. Data are expressed as mean ± standard deviation (SD), *n* = 8 (***p < 0.001 vs. Ctr; ^###^*p* < 0.001 vs. AFB1).

The TBARS assay was used to assess lipid peroxidation in kidney tissue. After 10 days of treatment, MDA levels were significantly increased by ~10-fold in broilers treated with AFB1 alone compared with the control group (^***^p < 0.0001 AFB1 vs. Ctr). CURC alone did not affect the kidney MDA levels, but significantly decreased to basal values the altered levels triggered by the AFB1 treatment (^###^*p* < 0.0001 AFB1 vs. AFB1 + CURC) (Figure 3).

### Effects of CURC and AFB1 on Oxidative DNA Damage

**Figure 4:**
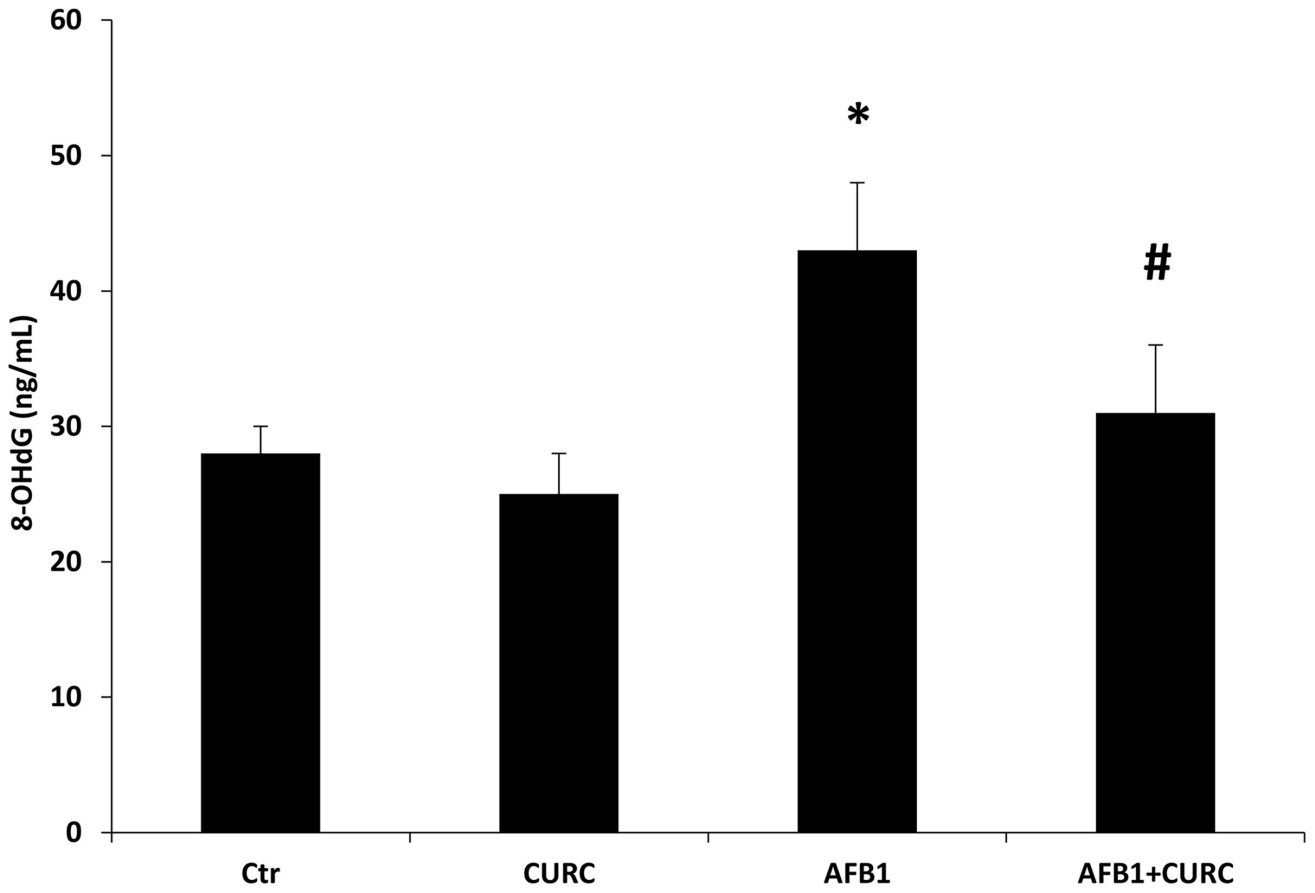
8-hydroxy-2′-deoxyguanosine (8-OHdG) levels measured in broiler serum samples at the end of the treatment (T10). Ctr, control group; CURC, Curcumin group; AFB1, Aflatoxin B1 group; AFB1 + CURC, Aflatoxin B1 group plus Curcumin. Data are expressed as mean ± standard deviation (SD), *n* = 8 (*p < 0.05 vs. Ctr; ^#^*p* < 0.05 vs. AFB1).

The oxidative DNA damage was examined by measuring the 8-OHdG levels in serum samples. At the end of the treatment, 8-OHdG levels were significantly higher in the AFB1 group with respect to the control group (^*^p < 0.05 AFB1 vs. Ctr) of ~30%. Such increase in DNA damage was significantly counteracted by CURC dietary supplementation in the AFB1-treated broilers (^#^*p* < 0.05 AFB1 vs. AFB1+ CURC) (Figure 4).

### Effects of CURC and AFB1 on NOX4 mRNA and Protein Expression

**Figure 5:**
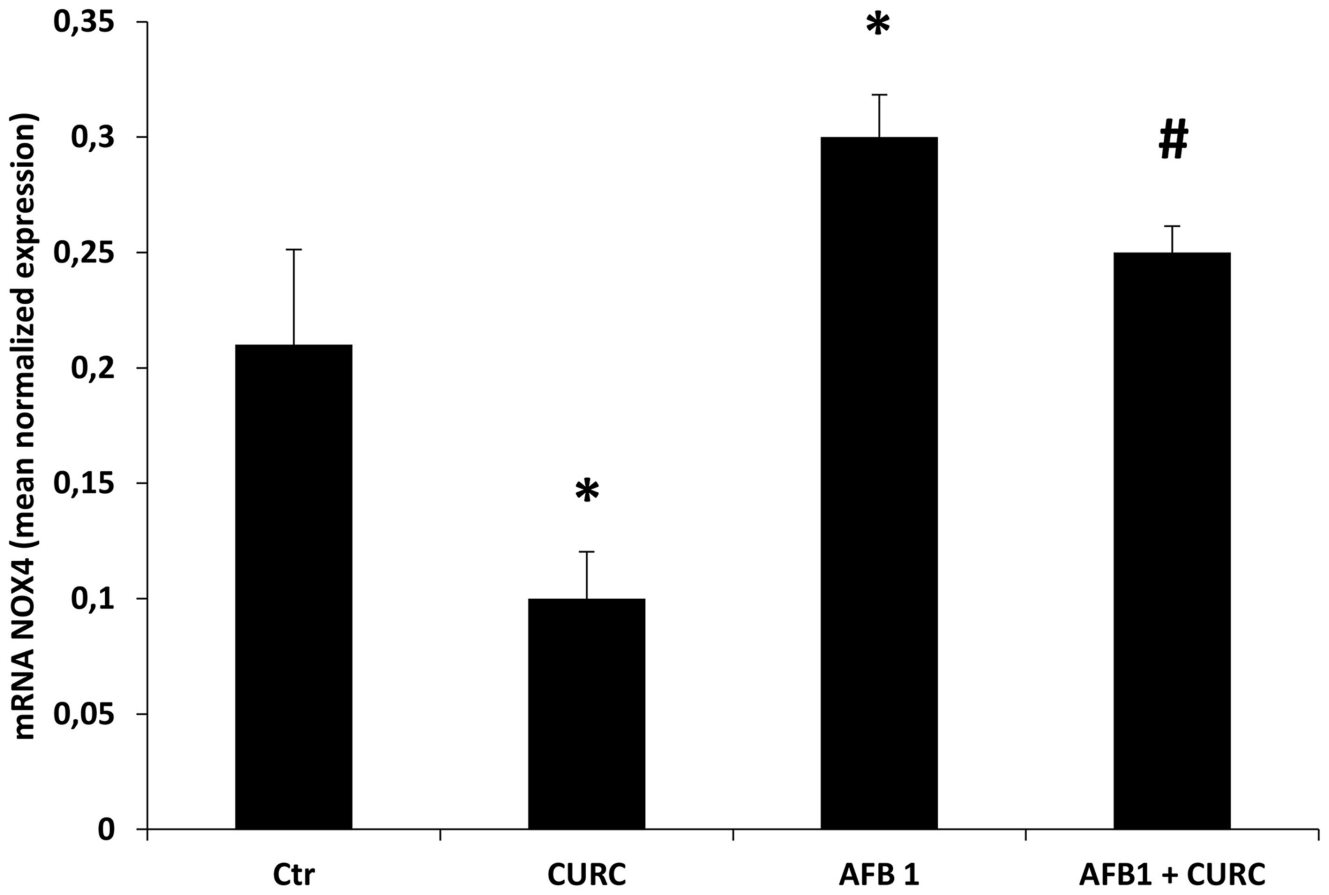
mRNA NOX4 levels in the kidney of broilers at the end of treatment (T10). Values are presented as mean normalized expression toward GAPDH expression. Ctr, control group; CURC, Curcumin group; AFB1, Aflatoxin B1 group; AFB1 + CURC, Aflatoxin B1 group plus Curcumin. Data are expressed as mean ± standard deviation (SD), *n* = 8 (*p < 0.05 vs. Ctr; ^#^*p* < 0.05 vs. AFB 1).

The mRNA and protein expression levels of NOX4 were investigated. Results showed that NOX4 mRNA content was significantly increased by almost 50% in broilers exposed to AFB1 alone, compared with the controls (^*^p < 0.05 AFB1 vs. Ctr). The supplementation of CURC in the AFB1-treated group induced a significant reduction of NOX4 expression to control values (^#^*p* < 0.05 AFB1 vs. AFB1 + CURC) (Figure 5).

**Figure 6:**
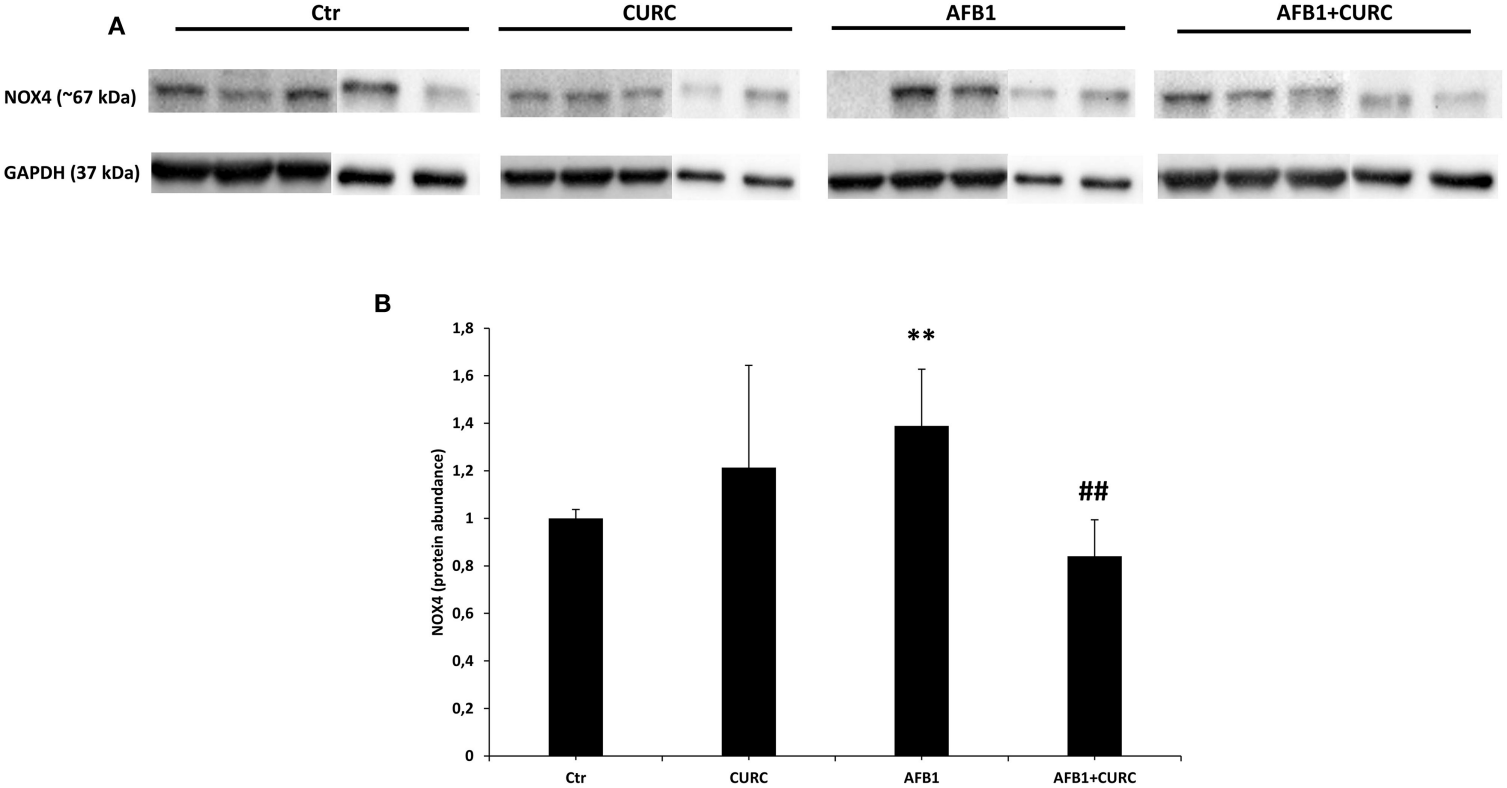
NOX4 protein expression in the kidney of broilers at the end of treatment (T10). **(A)** Representative western blot of NOX4. **(B)** Densitometric analysis of NOX4. Values were normalized toward GAPDH. Densitometric analysis is ex-pressed as arbitrary units. Ctr, control group; CURC, Curcumin group; AFB1, Aflatoxin B1 group; AFB1 + CURC, Aflatoxin B1 group plus Curcumin. Data are expressed as mean ± standard deviation (SD), *n* = 8 (**p < 0.01 vs. Ctr; ^##^*p* < 0.01 vs. AFB 1).

Such results were confirmed at protein level, where NOX4 expression (Figure 6) was significantly up-regulated in AFB1 treated animals with respect to the controls (AFB1 vs. Ctr). CURC supplementation restored NOX4 values to controls in AFB1-treated broilers (AFB1 vs. AFB1 + CURC).

## Discussion

It is well-known that AFB1 can cause severe health problems in chickens exposed to high levels of the mycotoxin (14). Diets contaminated with AFB1 cause functional problems in chicken liver and kidney (e.g., hepatic enzyme alterations, altered blood coagulation patterns, reduced renal protein content), and significantly inhibit egg production (14, 36). Moreover, studies performed in chicken and turkey spleen upon AFB1 exposure suggest that the mycotoxin affects the poultry immune system (37). To the best of our knowledge, few data are available about the health effects of AFB1 at low concentrations. The results of the present study revealed that feeding broilers with a diet contaminated by AFB1 at levels approaching the EU regulatory limits (0.02 mg/kg) is already able to induce an oxidative damage in the kidney. Moreover, the dietary supplementation of CURC effectively counteracted this toxic effect.

Although the AFB1-induced hepatotoxicity in broilers has been the subject of several investigations (16, 38), the mechanism of its renal toxicity is still unclear. Oxidative stress plays a major role in the toxic effects of AFB1 also in broilers, where the mycotoxin activates ROS generation and decreases the antioxidant defense in different tissues, such as liver, spleen, and heart (15, 16, 37). SOD, CAT, and GPx play a key role in the processes of detoxification by free radical and they protect the cell membrane from peroxidation; however, when the antioxidant defense system is not sufficient, changes in the antioxidant enzyme activity and MDA levels are observed (39, 40). Our results showed that SOD, CAT and GPx activities in the renal tissue of AFB1 treated chickens were decreased, while the administration of CURC was able to restore them to control values. Moreover, the lipid peroxidation of kidney tissue, measured by the TBARS assay, and the impairment of the blood antioxidant barrier (OXY test) revealed a significant prooxidant effect in the AFB1 group. Also in this case, the co-treatment with CURC restored both parameters to control values. Our results about the effects of AFB1 on the antioxidant system are in accordance with a study that demonstrated a slight reduction of SOD, CAT, and GPx activities, and an increase in lipid peroxidation in chick kidney upon AFB1 treatment, even if at much higher concentration (1 mg/kg) and for a longer period (45 days) (41). Similar results have also been reported in other animal species (42). Interestingly, the data of the present study demonstrate the protective effect of CURC against the oxidative damage in kidney induced by an AFB1 contaminated diet in chicken. Several research groups have shown the ability of CURC to counteract AFB1 hepatotoxicity in broilers (43, 44), but no data have been reported regarding the effect of such antioxidant on renal tissue, although the kidney is an AFB1 target organ. Thus, to the best of our knowledge, the present study is the first report revealing the beneficial effect of CURC on renal tissue of AFB1-exposed chicken. Although no tissue-specific comparisons can be made, the positive modulation of the antioxidant system triggered by CURC following AFB1 exposure in kidney resembles what has been reported for liver (43–45). Indeed, the decrease of SOD, CAT, and GPx activities, and the increase of MDA levels induced by AFB1 was prevented by CURC, as in the present study.

**Figure 7:**
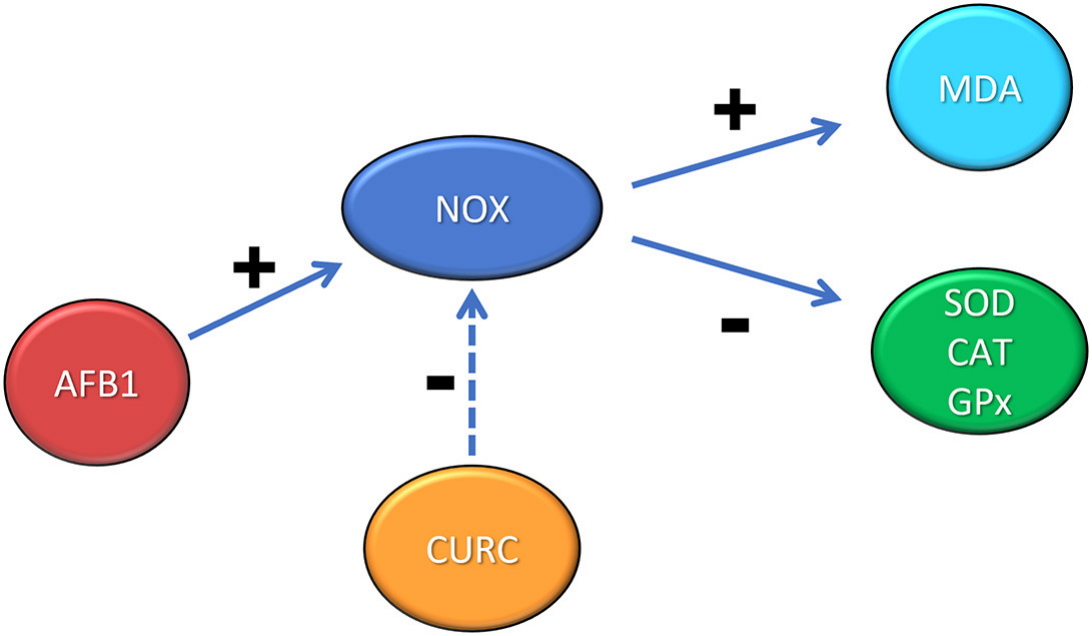
Schematic representation of the role of NOX in the renal oxidative stress induced by AFB1 and the protective effect exerted by CURC. +: positive modulation; −: negative modulation.

ROS generation induced by AFB1 modulates the inflammatory response activating the NOX pathway (46). NOX mediates several functions through redox signaling. Gp91-phox and p22-phox are two NOX subunits localized in integral membrane proteins; instead p47-phox, p40-phox, and p67-phox are located, in basal condition, in the cytosol. During stimulation, p47-phox undergoes phosphorylation and the complex is translocated to the membrane becoming active. As a result, O_2_ is converted to its successor products and becomes submerged in a toxic mixture of oxidants (47). The present study demonstrates the involvement of NOX system in the oxidative stress induced by AFB1 in chicken. In particular, mRNA levels of NOX were increased in AFB1 treated animals and CURC restored these levels. These results were confirmed at protein level, as the western blot analysis showed that NOX protein expression was significantly increased in AFB1 group and CURC reduced such augmentation to control values. These results suggest that the increase of lipid peroxidation and the decrease of SOD, CAT, and GPx activities are related to the NOX pathway activation, and that CURC may act as a NOX inhibitor, restoring both parameters (Figure 7). This finding is very interesting and can open new insights to counteract AFB1 toxicity in broilers fed contaminated diets, by using CURC as a NOX inhibitor.

In the literature, the assessment of 8-OHdG levels in different biological samples (e.g., urine, serum, and renal tissue) is used as a biomarker of oxidative damage in chronic kidney disease (48, 49). In the present work, we determined for the first time the serum levels of 8-OHdG in broilers dietary exposed to AFB1, and we showed the ability of CURC to prevent AFB1-induced DNA damage after 10-day treatment. Such results suggest that CURC may have a role in the reduced generation of the AFB1-epoxides that are primarily responsible for the generation of DNA-adducts, as already reported in liver (25, 44).

## Conclusion

Results from this study demonstrate that broiler kidney may be a target of AFB1-mediated oxidative stress even following the exposure to levels approaching the EU limits in feed. The mycotoxin caused a decrease of the enzymatic activity of SOD, CAT, and GPx, and an increase of MDA levels. Moreover, the NOX expression is upregulated at both mRNA and protein levels, showing a key role in the renal toxicity induced by AFB1. Interestingly, CURC is able to ameliorate all these deleterious effects, confirming to be an effective feeding strategy to prevent AFB1 toxicity in broilers. The effects of CURC on AFB1 hepatotoxicity and AFB1 disposition and fate in broilers from the present study will be the subject of a separate report.

## Data Availability Statement

The original contributions presented in the study are included in the article/supplementary material, further inquiries can be directed to the corresponding author/s.

## Ethics Statement

The study was conducted according to the guidelines of the Declaration of Helsinki and approved by the Institutional Animal Care and Ethic Committee of the University of Turin (Approval number = 319508/2017-PR).

## Author Contributions

SDam, FG, AS, PB, and CN conceived and designed the study. FG, PB, SDab, and AS performed the experiments. SDam, WJ, CLo, CLa, EA, and GA conducted the analysis. SDam and WJ wrote the original draft. FG, PB, and GA revised and edited the final version of the manuscript. RC, PB, and CN supervised the study. GA acquired the funding. All authors contributed to the article and approved the submitted version.

## Funding

This research was funded by H2020-EU.3.2. Integrated and innovative key actions for mycotoxin management in the food and feed chain (MycoKey) (ID n. 678781).

## Conflict of Interest

The authors declare that the research was conducted in the absence of any commercial or financial relationships that could be construed as a potential conflict of interest.

## Publisher's Note

All claims expressed in this article are solely those of the authors and do not necessarily represent those of their affiliated organizations, or those of the publisher, the editors and the reviewers. Any product that may be evaluated in this article, or claim that may be made by its manufacturer, is not guaranteed or endorsed by the publisher.
